# The Combination of Phylogenetic Analysis with Epidemiological and Serological Data to Track HIV-1 Transmission in a Sexual Transmission Case

**DOI:** 10.1371/journal.pone.0119989

**Published:** 2015-03-25

**Authors:** Min Chen, Yanling Ma, Chaojun Yang, Li Yang, Huichao Chen, Lijuan Dong, Jie Dai, Manhong Jia, Lin Lu

**Affiliations:** 1 Center for AIDS/STD Control and Prevention, Yunnan Center for Disease Control and Prevention, Kunming, Yunnan 650022, China; 2 College of Public Health, Kunming Medical University, Kunming, Yunnan 650500, China; Centers for Disease Control and Prevention, UNITED STATES

## Abstract

**Objective:**

To investigate the linkage of HIV transmission from a man to a woman through unprotected sexual contact without disclosing his HIV-positive status.

**Methods:**

Combined with epidemiological information and serological tests, phylogenetic analysis was used to test the *a priori* hypothesis of HIV transmission from the man to the woman. Control subjects, infected with HIV through heterosexual intercourse, from the same location were also sampled. Phylogenetic analyses were performed using the consensus *gag*, *pol* and *env* sequences obtained from blood samples of the man, the woman and the local control subjects. The *env* quasispecies of the man, the woman, and two controls were also obtained using single genome amplification and sequencing (SGA/S) to explore the paraphyletic relationship by phylogenetic analysis.

**Results:**

Epidemiological information and serological tests indicated that the man was infected with HIV-1 earlier than the woman. Phylogenetic analyses of the consensus sequences showed a monophyletic cluster for the man and woman in all three genomic regions. Furthermore, *gag* sequences of the man and woman shared a unique recombination pattern from subtype B and C, which was different from those of CRF07_BC or CRF08_BC observed in the local samples. These indicated that the viral sequences from the two subjects display a high level of similarity. Further, viral quasispecies from the man exhibited a paraphyletic relationship with those from the woman in the Bayesian and maximum-likelihood (ML) phylogenetic trees of the *env* region, which supported the transmission direction from the man to the woman.

**Conclusions:**

In the context of epidemiological and serological evidence, the results of phylogenetic analyses support the transmission from the man to the woman.

## Introduction

HIV undergoes fast genetic variation during its life cycle [1]. Although viral RNA sequences from individuals infected by a virus with the same origin are rarely identical, HIV strains from epidemiologically linked individuals are more similar than HIV strains from epidemiologically unassociated individuals [2, 3]. Therefore, combined with epidemiologic information, a potential HIV transmission linkage can be evaluated by comparing the degree of genetic similarity between HIV strains. In practice, phylogenetic analysis has been used to study the degree of relatedness between HIV genetic sequences, where the suspected transmission relationship or network can be supported [4–7].

The evolution of HIV in its host results in a cloud of genetic-related viral progenies, called quasispecies [8]. However, during transmission of HIV, only one or a few viruses are transmitted from source to recipient, which results in a reduced genetic variation in the recipient, a phenomenon known as the genetic bottleneck effect [9, 10]. The rapid evolution will increase HIV genetic diversity in the recipient. However, if the samples of the source and recipient are collected shortly after a transmission events, a subset of source viral sequences is more closely related to all recipient sequences than the other source sequences, which is displayed as the paraphyly of source viruses with respect to those of the recipient in the phylogenetic tree. Based on the paraphyletic relationship, the transmission direction can be inferred. Recently, the phylogenetic analysis of paraphyletic relationship was also used for source identification in criminal cases [11].

Standard Sanger sequencing is performed with PCR products obtained by amplifying the entire viral species extracted from a sample. However, Sanger sequencing does not reliably detect variants comprising less than 20% of the viruses in a sample, which means that its readout only represent a consensus sequence [12, 13]. To study the genetic characterization of viral quasispecies, single genome amplification (SGA) followed by direct sequencing of amplicon DNA is a necessary technique, which increases the potential to obtain multiple viral variants [14]. Thus, phylogenetic analysis with the sequences of viral quasispecies is important to investigate the viral genetic evolution in one individual.

In China, HIV is primarily transmitted by sexual contact, and the proportion of people living with HIV/AIDS (PLWHA) who were infected by this route is continually increasing [15]. Of the estimated new HIV infections, the proportion of sexual transmission increased from 75.7% in 2009 to 81.6% in 2011 [15]. As a measure of prevention and control, the newly diagnosed HIV-infected individuals are required to disclose HIV-positive status to their spouse or sexual partners, and are encouraged to engage in safer sexual behavior [16]. In China, taking the necessary precautionary measures to prevent others from being infected is the PLWHA’s obligation as defined by Regulations on AIDS Prevention and Treatment (2006) (P.R.C).

In this work, we report the use of phylogenetic analysis in a legal investigation of possible HIV transmission in China, where a man is suspected of transmitting HIV to a woman without disclosing his HIV status. Combining epidemiological and serological evidence, our results support that the virus was transmitted from the man to the woman.

## Materials and Methods

### Case information and samples

In February 2013, a 46-year-old woman residing in a city of Yunnan Province began to cohabit with a 47-year-old man who did not reveal his HIV-positive status. After the woman knew about the man’s HIV-positive status by chance, she attended the local voluntary counseling and testing site for HIV. The first blood sample of the woman was collected for HIV diagnosis on May 10, 2013 (sample F130510). HIV-1 infection status was determined by an enzyme immunoassay (Diagnostic Kit for Antibodies to Human Immunodeficiency Virus Type 1 and 2 (ElISA), bioMérieux sa, France) and confirmed by Western blot assay (HIV BLOT 2.2, MP Diagnostics, Singapore). On May 14, 2013, the woman was confirmed to be infected with HIV-1. Then, she accused the man of deliberately infecting her with HIV.

As part of the case investigation, the man's blood sample (M130805) and the woman's blood sample (F130805) were collected on August 5, 2013. The process of blood drawing and sample coding for each blood was witnessed by the local police officers. The samples were directly transported by the police officers to Yunnan Center for Diseases Prevention and Control (CDC). After verifying the coded identifier, CDC personnel re-coded the samples to blind the subjects’ identities and transferred them to lab assistants. To ensure that there was no cross-contamination, the man’s sample and the woman’s sample were separately handled. These two samples collected on August 5, 2013, were re-tested by Western blot for confirmation of HIV-1 infection. Based on similar studies [6, 7, 17, 18], the local controls (C01 to C18) were selected from individuals with similar epidemiological characteristics, including the same geographical area and risk group (heterosexual contact), and diagnosis around the time of the alleged transmission event (between January and March 2013). Information for these samples is shown in Table 1. After finishing all the molecular analyses, the original coded identifier were recovered for each sample. The local controls provided their written informed consent to participate in this study. The study was approved by Biomedical Ethics Review Committee of Yunnan Province.

**Table 1:** Samples Information.

Sample ID	Gender	Age	Sampling date	Reporting date
Subjects^1^				
M130805	Male	47	2013/8/5	2010/1/8
F130805	Female	46	2013/8/5	2013/5/14
F130510^2^			2013/5/10	
Controls				
C01	Female	25	2013/1/28	2013/2/1
C02	Male	50	2013/1/25	2013/2/1
C03	Male	35	2013/3/8	2013/3/8
C04	Male	30	2013/3/5	2013/3/15
C05	Male	25	2013/3/15	2013/3/15
C06	Female	67	2013/3/25	2013/3/29
C07	Male	39	2012/12/1	2013/1/18
C08	Male	49	2013/1/3	2013/1/18
C09	Female	34	2013/1/10	2013/1/18
C10	Female	34	2013/1/11	2013/1/18
C11	Female	22	2013/1/4	2013/1/18
C12	Male	30	2013/1/4	2013/1/18
C13	Male	31	2013/1/10	2013/1/18
C14	Female	58	2013/2/15	2013/2/27
C15	Male	44	2013/2/16	2013/2/22
C16	Male	38	2013/2/18	2013/2/22
C17	Female	56	2013/2/18	2013/2/22
C18	Male	31	2013/2/18	2013/2/22

1. All samples were infected through heterosexual contact.

2. F130805 and F130510 are from the same person.

### RNA extraction and cDNA synthesis

Viral RNA was extracted from 140 μl of plasma using the QIAamp Viral RNA Mini kit (Qiagen, Valencia, CA, United States) according to the manufacturer’s instructions. RNA was recovered from the spin columns in a final elution volume of approximately 50 μl. Reverse transcription of 20 μl RNA to single-stranded cDNA was performed in 50μl reaction volumes with SuperScript III (Invitrogen Life Technologies, Carlsbad, CA) according to the manufacturer’s instructions.

### The amplication of consensus sequences of gag, pol and env gene

RNA samples were directly subjected to nested polymerase chain reactions (PCR) to generate fragments of *gag* (HXB2: 781–1861; encoding portions of p17 and p24), *pol* (HXB2: 2147–3462; encoding the protease and the first 299 residues of reverse transcriptase) and *env* (HXB2: 7002–7541, encoding the V3∼V4 region). The first PCR reaction was performed using One Step reverse transcription PCR Kit (Takara, Dalian, China). The second PCR reaction was performed using 2×Taq PCR Master Mix (Tiangen, Beijing, China). The details of primers and PCR reaction conditions were previously described [19]. The products were analyzed using 1% agarose gel electrophoresis. Positive samples were separately sent to ZIXIBIO Co. (Beijing, China) for sequencing using an ABI 3730XL automated DNA sequencer (Applied Biosystems, Carlsbad, USA) with the following primers: *gag*: GUX/GDX; *env*: 33F/48R; *pol*: PROS3, RTAS, RTB, PROC1S, and RT20S3. The details of sequencing primers were previously described [19].

### Single genome amplification and sequencing (SGA/S)

According to the Poisson distribution, cDNA was serially diluted and amplified in replicate to identify the dilution yielding PCR success rates of <30% at which point the majority of amplicons are derived from a single copy template [12]. In detail, cDNA was serially diluted by 1:10 and 1:100. Each dilution was used in 16 wells for PCR, and if a dilution yielded product in 4 wells, then that dilution was used for a full 96-well plate. If none of the dilutions yielded 4 positive reactions, the dilution of cDNA was adjusted and PCR repeated until the appropriate dilution was found. First-round PCR for *env* region was carried out with 2.5 μl diluted cDNA in a 25 μl reaction mixture containing ExTaq polymerase (Takara, Dalian, China). 5 μl of product from the first-round PCR was subjected to second-round PCR in a volume of 50 μl. The same primers for consensus *env* gene amplification were used. The final products were sequenced.

### Sequence Analysis

The assembly of the different sequences generated from the same gene region of each sample was performed using the DNA sequence analysis software Sequencher 5.0 (Gene Codes, Ann Arbor, MI). ClustalW Multiple alignment and manual editing were performed using Bio-Edit 7.0 software. The reference sequences were obtained from the NIH/NIAID-funded HIV Databases (http://hiv-web.lanl.gov/content/index), covering the major HIV-1 subtypes/CRFs.

Genetic distance calculations and phylogenetic analyses were performed with MEGA (Molecular Evolutionary Genetics Analysis, version 6.0) [20]. The genetic distances of consensus sequences from the woman and local controls to that of the man were calculated for the *gag*, *pol* and *env* fragments using the Kimura two-parameter model. Phylogenetic tree analyses were performed using the neighbor-joining method based on the Kimura two-parameter model with 1000 bootstrap replicates. The genetic distances of SGA-derived *env* lineages from the man, woman and two controls (C01 and C02) were calculated using the Kimura two-parameter model.

To demonstrate possible intersubtype mosaicism, candidate sequences were analyzed using the Recombination Identification Program (RIP, version 3.0; http://hiv-web.lanl.gov) and were further analyzed with similarity plot analyses using Simplot (version 3.5.1; S. Ray, Johns Hopkins University, Baltimore, MD)[21].

### Bayesian MCMC evolutionary analysis

Phylogenetic analysis of quasispecies for *env* was performed using Bayesian Markov chain Monte Carlo (MCMC) method. The general time reversible (GTR) model plus a gamma distribution (Г4) among site rate heterogeneity (I) model (GTR+I+Г4) was evaluated as the best nucleotide substitution model for all datasets by the jModeltest version 2.1.2. Bayesian MCMC analyses were performed using a Bayesian uncorrelated exponential relaxed molecular clock method in combination with three different coalescent tree priors (‘Constant Size’; ‘Exponential Growth’ and ‘Bayesian Skyline’) under the selected nucleotide substitution model in the BEAST v1.7.4 package [22]. Each MCMC analysis was run for at least 10 million generations and sampled every 1,000 generations. The resulting log-files were analyzed in Tracer v1.5 and the Bayes Factor was calculated to compare molecular clock models, using marginal likelihood as implemented in Tracer v.1.5. Based on marginal likelihood, the uncorrelated exponential relaxed molecular clock model with Bayesian Skyline coalescent tree priors was selected as the best model. The Maximum Clade Credibility (MCC) tree was obtained by TreeAnnotator v1.7.4 with a burn-in of the initial 25% of generated trees, and examined by FigTree V1.3.1, which was also used to estimate the evolutionary rates and the dates to tMRCA of various nodes on the MCC tree.

### Sequence Data

All the sequences obtained in this study were submitted to GenBank under accession numbers KM370170 to KM370303.

### Statistical Analysis

Statistical analyses were conducted using Prism 6 (GraphPad Software Inc. La Jolla, CA). Data were expressed as mean (minimum-maximum). When comparing the genetic distances of consensus sequences, Wilcoxon signed-rank test was applied. When comparing the intraperson and interperson genetic distances of viral quasispecies, Kruskal-Wallis test was applied. A *p*-value <0.05 was considered statistically significant.

## Results

### Epidemiological information and Serological Testing

According to the record of China Information System for Diseases Control and Prevention, the man had been reported to be HIV-positive on January 8, 2010. However, the woman was reported as a HIV-infected individual on May 14, 2013. The western blot results showed only three HIV-specific antibodies (anti-p24, anti-gp120 and anti-gp160) in the sample drawn from the woman on May 10, 2013 (F130510). On August 5th, more HIV-specific antibodies (anti-p24, anti-p31, anti-gp41, anti-p51, anti-p66, anti-gp120, anti-gp160) were detected in her sample (F130805), indicating a progression of the immune response to HIV-1, suggesting a recent infection.

### Phylogenetic analysis of consensus sequences

For each sample, partial *gag* gene, *pol* gene, and *env* gene were amplified and sequenced. To examine the relationships among predominant HIV-1 strains within the man and the woman, paired genetic distances from sequences of the woman and local controls to those of the man were calculated within *gag*, *pol* and *env* regions, respectively (Table 2). For the woman, the genetic distances to the man’s virus were 0.0077 in *gag*, 0.0111 in *pol* and 0.0040 in *env*. For the local controls, the genetic distances to the man’s virus were 0.0488–0.1934 in *gag*, 0.0203–0.1386 in *pol*, and 0.1220–0.3395 in *env*. Within all three gene regions, the mean genetic distances of the woman’s virus (F130510 and F130805) to the man’s (M130805) were statistically smaller than those of the controls’ combined (C01-18) to the man’s (M130805) (p<0.05).

**Table 2:** The genetic distances to M130805 within three genes regions.

Sample ID	Genotyping by *gag*	Genetic Distances in *gag* region	Genotyping by *pol*	Genetic Distances in *pol* region	Genotyping by *env*	Genetic Distances in *env* region
M130805	BC	-	CRF08_BC	-	C	-
F130805	BC	0.0077	CRF08_BC	0.0111	C	0.0040
F130510	BC	0.0077	CRF08_BC	0.0111	C	0.0040
C01	CRF08_BC	0.0550	CRF08_BC	0.0418	C	0.1389
C02	CRF08_BC	0.0532	CRF08_BC	0.0275	C	0.1220
C04	CRF08_BC	0.0738	CRF08_BC	0.0328	C	0.1382
C05	CRF08_BC	0.0499	CRF08_BC	0.0347	C	0.1617
C06	CRF08_BC	0.0639	CRF08_BC	0.0304	C	0.1700
C07	CRF08_BC	0.0672	CRF08_BC	0.0209	C	0.1321
C08	CRF08_BC	0.0488	CRF08_BC	0.0203	C	0.1774
C09	CRF08_BC	0.0674	CRF08_BC	0.0388	C	0.1439
C10	CRF08_BC	0.0621	CRF08_BC	0.0210	C	0.1251
C11	CRF08_BC	0.0565	CRF08_BC	0.0303	C	0.2462
C12	CRF08_BC	0.0566	CRF08_BC	0.0266	C	0.2020
C14	CRF08_BC	0.0695	CRF08_BC	0.0384	C	0.1765
C16	CRF08_BC	0.0488	CRF08_BC	0.0341	C	0.1396
C18	CRF08_BC	0.0551	CRF08_BC	0.0274	C	0.1482
C03	CRF01_AE	0.1710	CRF01_AE	0.1376	CRF01_AE	0.2644
C13	CRF01_AE	0.1934	CRF01_AE	0.1386	CRF01_AE	0.3332
C15	CRF01_AE	0.1612	CRF01_AE	0.1266	CRF01_AE	0.2928
C17	CRF01_AE	0.1839	CRF01_AE	0.1253	CRF01_AE	0.3395

**Fig 1:**
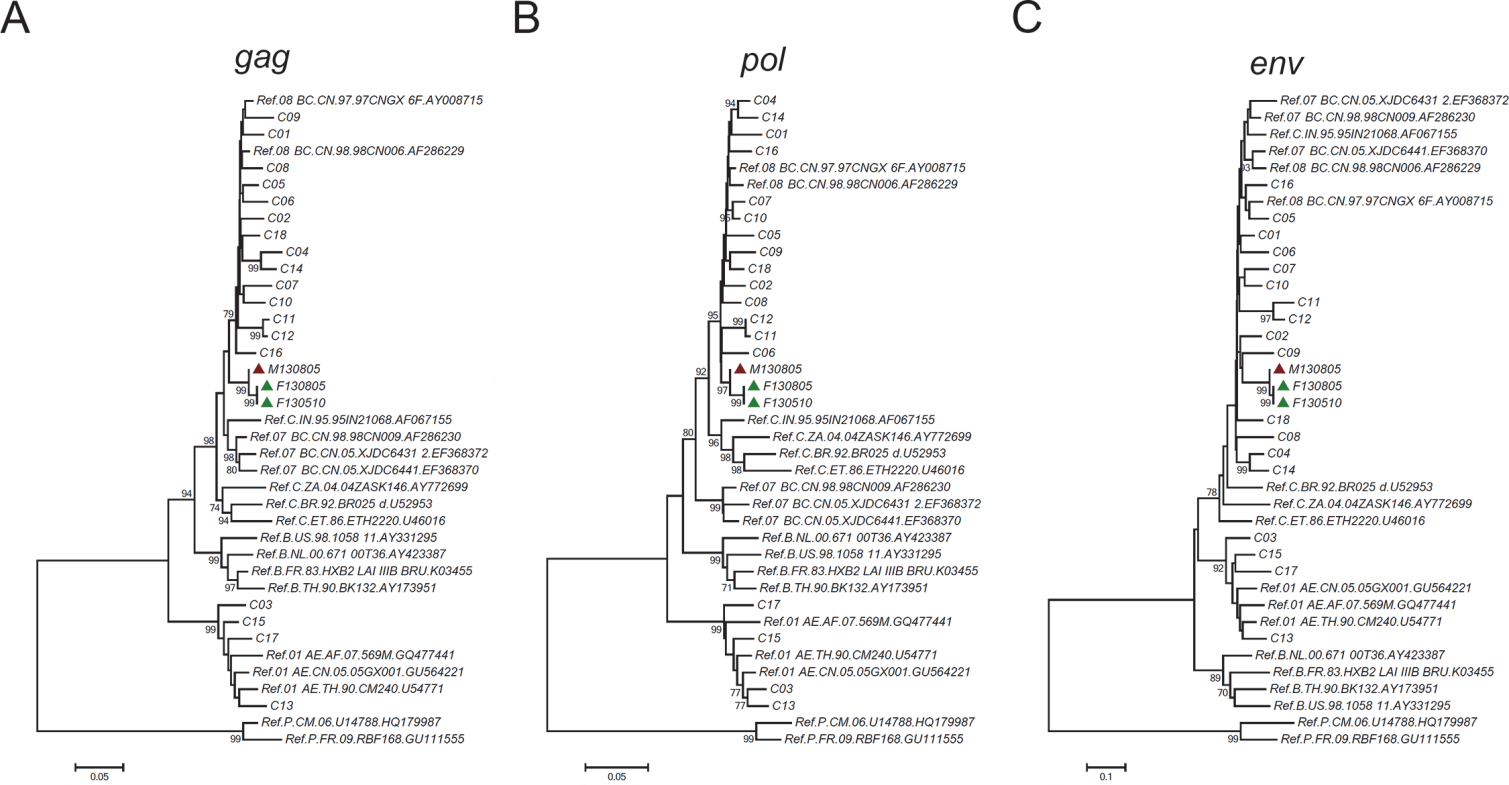
Neighbor-joining phylogenetic tree of consensus sequences from the man, the woman and local controls. A, Neighbor-joining phylogenetic tree for consensus *gag* sequences and reference sequences. The scale bar indicates 5% nucleotide sequence divergence. B, Neighbor-joining phylogenetic tree for consensus *pol* sequences and relative reference sequences. The scale bar indicates 5% nucleotide sequence divergence. C, Neighbor-joining phylogenetic tree for consensus *env* sequences and relative reference sequences. The scale bar indicates 10% nucleotide sequence divergence. Values on the branches represent the percentage of 1000 bootstrap replicates and bootstrap values over 70% are shown in the tree. Red triangle: the sequence from the male source; Green triangle: the sequences from the female recipient.

**Fig 2:**
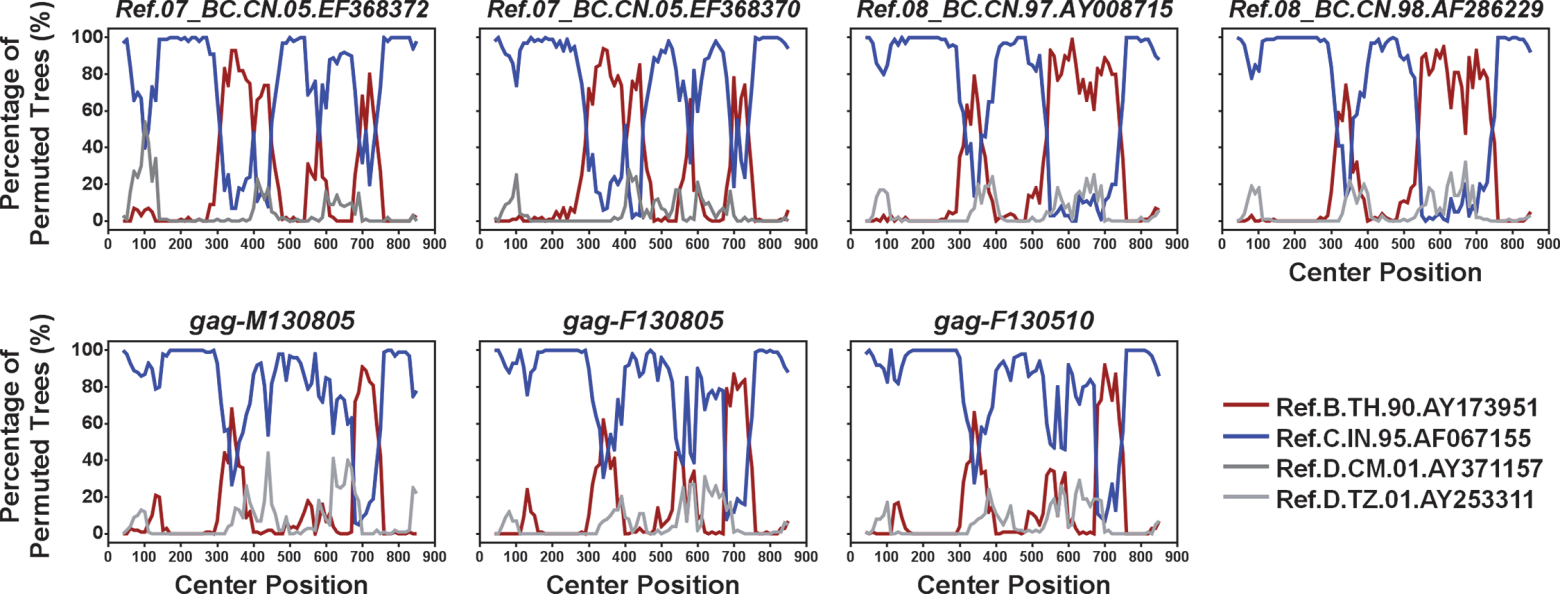
Bootscanning analysis of possible intertype mosaicism. Window: 80 bp, step: 10 bp, GapStrip: on, reps: 100, Kinura (2-parameter), T/t: 2.0. The reference sequences are shown at the bottom right of the figure.

Phylogenetic analysis was further used to examine the genetic relatedness between the viruses of the man and the woman. As shown in the neighbor-joining phylogenetic trees of *gag*, *pol* and *env* (Fig. 1), the woman’s sequences, but not the local controls’, clustered with the man’s with high bootstrap values (99%), which strongly supported the genetic relatedness between the woman’s strain and the man’s. However, in the phylogenetic tree of *gag*, the cluster of the woman and the man lay outside of the clades of subtypes/Circulating Recombinant Forms (CRFs), suggesting a new recombination event. Bootscanning analyses confirmed that *gag* sequences of the woman and the man had the same recombination pattern from subtype B and subtype C, which was different from those of CRF07_BC and CRF08_BC (Fig. 2). This also suggested that the strain infecting the woman was closely related to that infecting the man.

### Phylogenetic analysis of viral quasispecies

The quasispecies of *env* within the man, the woman and two local controls were obtained by single gene amplification (SGA). First, the mean genetic distances for viral sequences from the same sample were calculated (Table 3). Among them, the mean genetic distance of the viral sequences from F130805 was less than that from M130805 (p<0.05), that is, the variability of the quasispecies within the woman’s strain was smaller, which suggested she had been infected with HIV for a shorter time. Furthermore, the mean genetic distances to the man’s viruses were calculated (Table 3). For the woman, the mean genetic distance to the man’s viruses was 0.0430, which was within the range reported for known epidemiologically linked infections [2, 3, 23, 24]. For controls C01 and C02, the mean distances to the man’s viruses were 0.1522 and 0.1544, which were typical of measured distances for viruses from epidemiologically unlinked infections [2]. Furthermore, the genetic distance of the woman’s quasispecies to the man’s was statistically smaller than that of the C01’s or C02’s to the man’s.

**Table 3:** The genetic distances of *env* quasispecies.

Sample ID	Clones	Intraperson genetic distance	Interperson genetic distance
			To M130805
M130805	22	0.0581 (0.0000–0.1890)#	-
F130805	21	0.0060 (0.0000–0.0120)	0.0430 (0.0080–0.1920)
C01	12	0.0259 (0.0000–0.0710) #	0.1522 (0.1230–0.2060)*
C02	16	0.0276 (0.0000–0.0570) #	0.1544 (0.1310–0.2060)*

Data were expressed as mean (minimum-maximum).

#P<0.05, when comparing with the intraperson genetic distance of the viral sequences from F130805.

*P<0.05, when comparing with the interperson genetic distances of F130805 to M130805.

**Fig 3:**
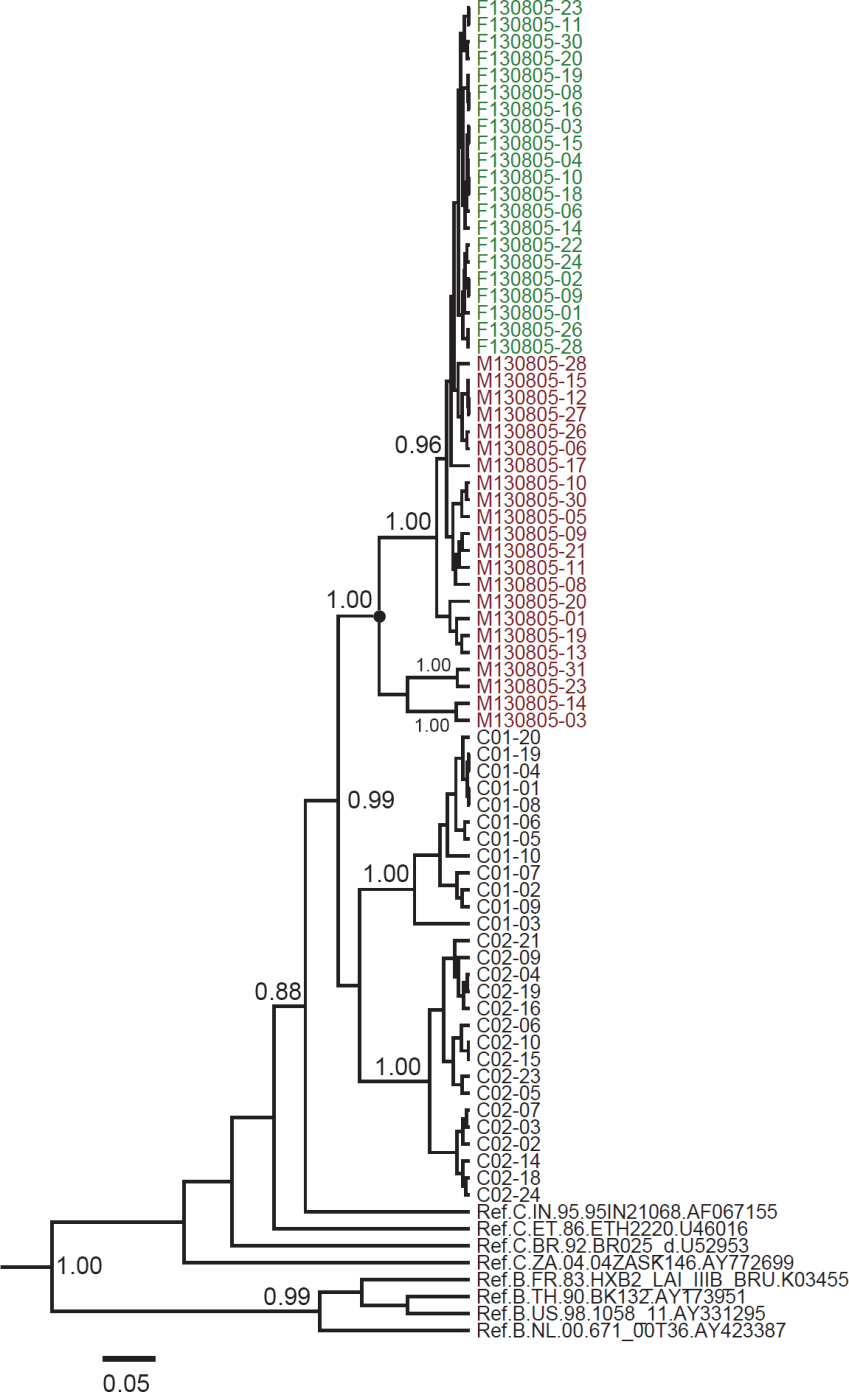
Maximum clade credibility (MCC) tree for *env* quasispecies from the subjects. The MCC trees were obtained by Bayesian MCMC analysis based on partial *env* gene (HXB2: 7002–7541) implemented in BEAST. The uncorrelated exponential relaxed molecular clock method was used in combination with the Bayesian Skyline coalescent tree prior under GTR+I+G4 nucleotide substitution model. Red taxon: the viral quasispecies from the man; Green taxon: the viral quasispecies from the woman. The posterior probabilities of the key nodes are indicated. The most recent common ancestor of sequences from the man is shown by a filled circle. The scale bar indicates 5% nucleotide sequence divergence.

If a paraphyletic relationship is observed in the phylogenetic tree, the direction of transmission can be inferred. The quasispecies of *env* gene from the man and the woman were analyzed with Bayesian MCMC method. In maximum clade credibility (MCC) tree for the *env* gene (Fig. 3), with the exception of M130805-03, M130805-14, M130805-23 and M130805-31, the other sequences from M130805 clustered with all sequences from F130805, showing that viral sequences from M130805 were paraphyletic with those from F130805. The result was also confirmed by maximum-likehood method (S1 Fig.). Thus, we inferred that the direction of transmission occurred from M130805 to F130805.

## Discussion

In this study, we used phylogenetic analyses to test the *a priori* hypothesis of HIV-1 transmission from a male source to a female recipient. The *a priori* hypothesis was raised based on epidemiological and serological information. First, the man was reported to be HIV-positive three years earlier than the woman; second, the woman was a recent HIV-1 infector, supported by the increase of HIV-1 specific antibodies detected in the first three months after diagnosis; third, they had a history of cohabitation, a fixed sexual relationship. Under this circumstance, if the genetic relatedness between the viruses from the two individuals is confirmed, the transmission relationship from the man to the woman can be established. As shown in the results, the consensus sequences from the man and woman formed a monophyletic cluster in all three genome regions when using phylogenetic analyses. Furthermore, as a distinct marker, *gag* sequences of the man and woman shared a unique BC recombination pattern, which was different from those of CRF07_BC and CRF08_BC, the latter of which was the predominant genotype in the local geographic region [25]. This indicates that the viral sequences from the two subjects display a high level of similarity and are more closely related to each other than to other strains circulating in the local area. Further, as molecular evidence for the direction of transmission, the paraphyly of the man’s sequences with respect to the woman’s sequences was observed, which suggests that the man was the source of infection. Thus, both the relatedness and the direction of transmission were proved by phylogenetic analysis, which strongly support the epidemiological linkage.

DNA fingerprinting is a widely-accepted technique employed by forensic scientists as a means of matching individuals’ respective DNA profiles [26]. Unlike DNA fingerprinting, phylogenetic analysis is a technique to compare how closely DNA sequences from different sources are related [27]. When using HIV phylogenetic analysis for forensic purpose, investigators are often warned to be cautious [18]. In fact, even if the strains carried by two subjects are more related to each other than the control strains, the following circumstances could not be excluded: both the two subjects were infected by one or more third parties with similar viruses, or a third party mediated HIV transmission from one subject to the other. Thus, the use of phylogenetic analysis should be careful. Phylogenetic evidence, in the context of other clinical and epidemiological evidence, can provide support for linkage between cases, but cannot be proof of transmission by itself [18]. In this investigation, we provide epidemiological and serological evidence of possible transmission between the two subjects prior to phylogenetic testing.

Apart from relatedness between HIV samples, transmission direction is also important for transmission tracing. In the present case, the transmission direction was effectively proved using phylogenetic analysis of pharaphyletic relationship. However, a paraphyletic relationship will gradually decline over time due to lineage extinction, the elimination of some strains by the host’s immune system or antiretroviral therapy [1, 28]; or due to the rapid evolution of HIV. Thus, the time lag between transmission and sampling is critical for the effectiveness of the analysis for paraphyly. In most cases, the start of a legal investigation is long after the occurrence of transmission, which could compromise the outcome of the analysis. To reduce the influence of long time lag on the prediction of transmission direction, Yang et al. developed a novel approach based on the bottleneck effect and co-receptor switching [29]. HIV entry into target cells is mediated by CD4 and one of two chemokine receptors, CCR5 or CXCR4 [30, 31]. Viruses using CCR5 as co-receptor are generally present in primary infections, whereas viruses using CXCR4 co-receptor emerge during later stages of infection, which is called as co-receptor switching [32]. In the novel approach, a group of nonsequential but related amino acids within a fixed-length window is defined as a “pattern”. “Common patterns” are those that appear in both R5 and non-R5 sequences. The number of common patterns will decrease from donor to recipient because of the bottleneck effect. Compared with phylogenetic analysis, this approach performed better on transmission pair with long time lags. However, the total number of common patterns is determined by the size of the available dataset and may change as the virus evolves. Unlike phylogenetic analysis, there is no available software for this approach.

Although the application of phylogenetic analysis should be particularly cautious, in this study, we integrated phylogenetic analysis with epidemiological and serological data to investigate HIV transmission between two individuals. The purpose is to improve the reliability of phylogenetic analysis. The molecular data in the current investigation is sufficient to support the epidemiological conclusion that the man transmitted HIV to the woman.

## Supporting Information

S1 Fig Maximum-likelihood phylogenetic tree of SGA-derived *env* lineages from the man, woman and two local controls. Phylogenetic analysis of quasispecies for *env* was performed using the maximum-likelihood method based on the Kimura two-parameter model with 1000 bootstrap replicates. Values on the branches represent the percentage of 1000 bootstrap replicates. Red triangle: the viral quasispecies from the man; Green triangle: the viral quasispecies from the woman.
(TIF)
