# Supplementary figures and images for: The Combination of Phylogenetic Analysis with Epidemiological and Serological Data to Track HIV-1 Transmission in a Sexual Transmission Case
Source: PLoS One. 2015 Mar 25;10(3):e0119989. doi: 10.1371/journal.pone.0119989 (PMC4373787; doi:10.1371/journal.pone.0119989)

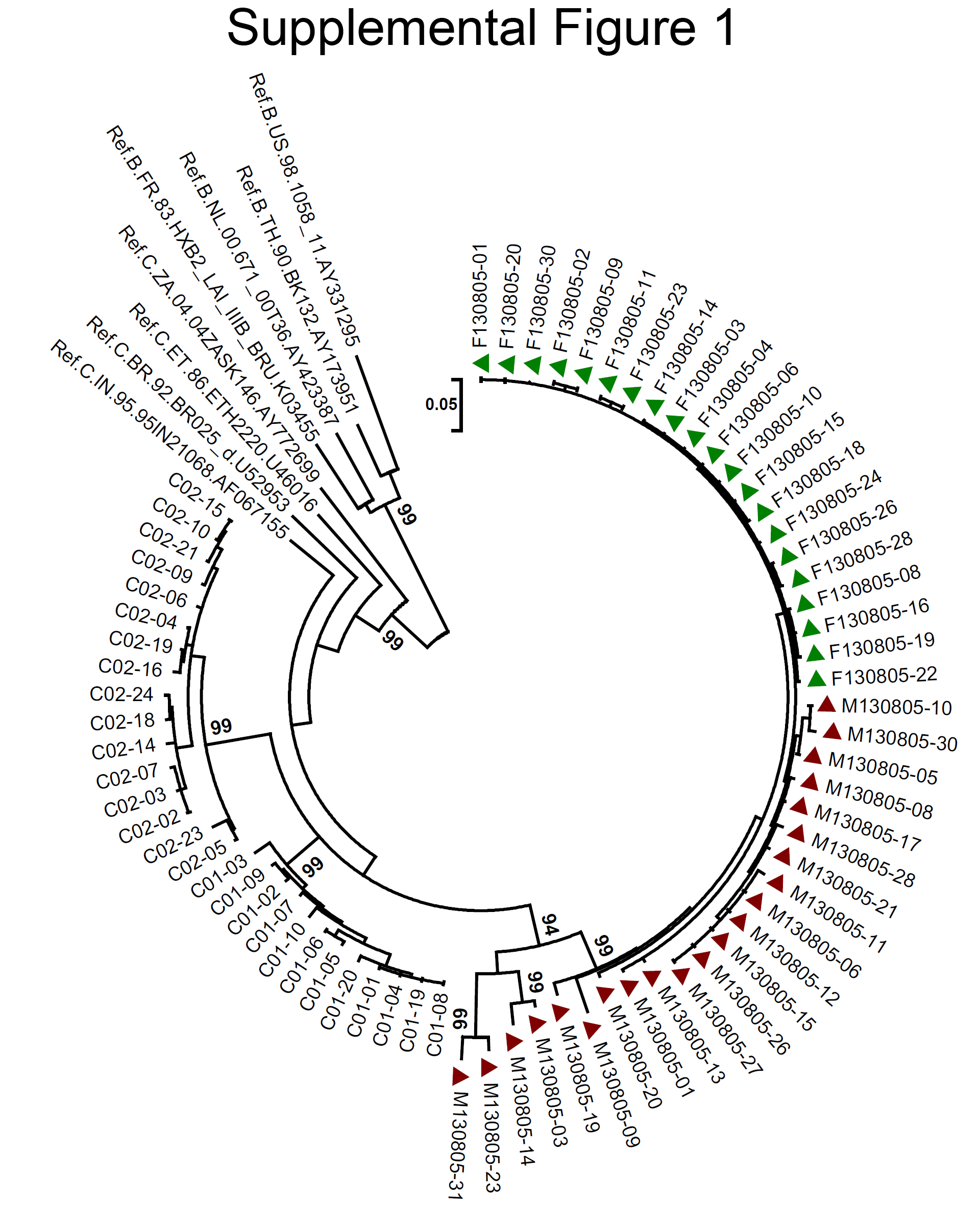

Supplement: S1 Fig — Phylogenetic analysis of quasispecies for env was performed using the maximum-likelihood method based on the Kimura two-parameter model with 1000 bootstrap replicates. Values on the branches represent the percentage of 1000 bootstrap replicates. Red triangle: the viral quasispecies from the man; Green triangle: the viral quasispecies from the woman. (TIF) [file pone.0119989.s001.tif]
